# Health service use of infants involved in family justice care and supervision proceedings in Wales: a longitudinal national data linkage study.

**DOI:** 10.23889/ijpds.v7i3.1989

**Published:** 2022-08-25

**Authors:** Ian Farr, Carys Jones, Laura Cowley, Bachar Alrouh, Linda Cusworth, Ashley Akbari, Stefanie Doebler, Karen Broadhurst, David Odd, Lucy Griffiths

**Affiliations:** 1 Swansea University; 2 Lancaster University; 3 Cardiff University

## Objectives

When an infant is identified as having suffered or is at risk of suffering significant harm from parents or caregivers, section 31 care and supervision proceedings (s.31, Children Act 1989) may be issued. We examined the healthcare use of infants under one year old subject to these proceedings in Wales.

## Approach

A retrospective e-cohort study utilising data held in the Secure Anonymised Information Linkage (SAIL) Databank. General Practice records, emergency department attendances, and hospital admissions (non-elective and elective) were linked with family justice (Cafcass Cymru) data between 2011–2020 for all s.31 proceedings (n = 920). By comparing to the infant population not undergoing family law proceedings (n = 18179), regression models examined the incidence and rate of healthcare events between birth and the court proceeding application date. Wider determinants of health were sequentially added (infant perinatal factors, maternal mental health, sex, area-level deprivation). Reasons for healthcare events were also investigated.

## Results

These models showed that infants who were subject to s.31 care and supervision proceedings were more likely to have required healthcare prior to proceedings than the comparison group. A similar pattern was shown for the rate of healthcare events. Even when wider determinants of health were included in the models, this difference was especially pronounced for incidence and event rate ratios for emergency department attendances, [incidence RR = 1.73, CI = 1.52–1.96; event RR = 2.08, CI = 1.82–2.38] and non-elective inpatient admissions [incidence RR = 2.91, CI = 2.57–3.28; event RR = 3.84, CI = 3.31–4.45]. Infants in s.31 proceedings were more likely to require healthcare for injury and poisoning, and other external causes.

## Conclusion

This is the first population-wide evidence on the health of infants subject to s.31 care and supervision proceedings in Wales. These findings highlight the increased healthcare utilisation for this population. The study helps to build a better understanding of the needs and vulnerabilities of infants in the family justice system.

